# Improvements in social functioning reported by a birth cohort in mid-adult life: A person-centred analysis of GHQ-28 social dysfunction items using latent class analysis

**DOI:** 10.1016/j.paid.2006.07.010

**Published:** 2007-01

**Authors:** George B. Ploubidis, Rosemary A. Abbott, Felicia A. Huppert, Diana Kuh, Michael E.J. Wadsworth, Tim J. Croudace

**Affiliations:** a Department of Psychiatry, University of Cambridge, Box 189, Addenbrooke’s Hospital, Hills Road, Cambridge CB2 2QQ, UK; b MRC National Survey of Health and Development, Royal Free & University College Medical School, Department of Epidemiology and Public Health, 1–19 Torrington Place, London WC1E 6BT, UK

**Keywords:** Latent structure analysis, General health questionnaire, Positive outcomes, Social dysfunction, Positive functioning, Latent class analysis

## Abstract

The General Health Questionnaire is widely used to measure the health status of individuals. Most studies have focused on traditional score values for one or more dimensions of psychopathology. We introduce a new analysis model that is person-centred and uses a latent structure approach to group individuals by a discrete latent variable. Data were drawn from a midlife (age 53) follow up of a national birth cohort study (*n* = 3035). For both men and women, three groups (latent classes) were sufficient to summarise individuals’ reports of recent changes in social functioning. The groups differed in the number and nature of the reported changes. Furthermore, they were shown to differ in terms of: (1) reported general health, (2) in mean scores on the conventional GHQ factors and (3) in several other variables external to the GHQ (happiness in job, ability to express feelings and self-confidence). Latent Class Analysis of positively worded GHQ items defined groups who differ in perceptions of recent positive changes in social functioning. These groups extend the value of individual health profiles afforded by the GHQ by using distinctions between categories in the first and second responses that are usually combined.

## 1 Introduction

The 28 item version of the General Health Questionnaire (GHQ-28; Goldberg & Hillier, 1979) is the most widely used screening instrument for detecting minor psychiatric disorders in community samples. The GHQ offers a continuous measure of psychological distress or current mental health status that captures the probability of having a current disorder, and also predicts imminent onsets. The 28 item “scaled” version describes individual health status in terms of four dimensions of psychological morbidity and social functioning: (A) somatic symptoms, (B) anxiety/insomnia, (C) social dysfunction and (D) severe depression. These are rated on four point rating scales [1–2–3–4]. Traditional use of the GHQ often relies on sum scores (Werneke, Goldberg, Yalcin, & Ustun, 2000) using Likert [1–2–3–4], traditional/binary [0–0–1–1] or chronicity scoring ([0–0–1–1] for positive and [0–1–1–1] for negative items). All analyses to date have been from a variable-centred perspective, emphasising scaling and scoring, rather than classification, and do not consider or entertain any person centred analysis approach (Muthen & Muthen, 2000).

In its general mode of use, the GHQ-28 is not intended to measure positive attributes only the absence of distress (Lewis, 1992). Innovative approaches to coding positive responses to positively worded GHQ items (in the GHQ-30) have been suggested before and have been offered as a candidate model for scoring aspects of positive mental health as a continuum (Huppert & Whittington, 2003). Our interest here is in characterising a person-centred approach to GHQ28 item data using the 1–0–0–0 response coding to the positively worded GHQ28 items. We therefore make use of distinctions between responses in the first two categories of the four point Likert response scale that are usually combined.

We introduce a typological (latent structure) approach to analysis of responses to GHQ items. Our aim is to identify groups within the general population who report different subjective perceptions of *positive* change in social functioning. We view social functioning as a dimension of social well-being, and wish to identify groups who reported improvements in the recent past. According to Keyes (1998), as adults age they encounter tasks that force them to choose and adapt within a social environment, which is a major life change with distinct consequences for judging a life well-lived. Social well-being can therefore be conceptualized as the appraisal of ones circumstances and functioning in society (Keyes, 1998), or through personal evaluation of task performance. Epidemiological studies, that sample individuals from the general population, should therefore investigate patterns of social functioning, since this is likely to be a component of optimal mental health.

Furthermore, from a clinical perspective, a deterioration in social functioning is often a characteristic of impending common mental disorders such as depression or anxiety, or more severe illnesses such as bipolar disorder, schizophrenia or other psychoses (DSM-IV-TR; American Psychiatric Association, 2000). Consequently, a dynamic assessment of social functioning such as the positive change in social functioning emphasised here, may be a useful complement to any clinical assessment battery applied by researchers in clinical or consulting settings, especially in interventions that have been designed to improve social functioning as a component of mental health.

We therefore applied Latent Class Analysis (LCA) (Lazarsfeld & Henry, 1968; McCutcheon, 1987) to positive responses to items from the social dysfunction subscale of the GHQ-28. We justify this use of LCA by our main interest in *grouping* individuals (using a person centred approach), rather than *scoring* individuals along a continuum of positive social functioning. Dimensional/continuum models could be derived using a form of factor analysis or Item Response Theory (cf. latent trait modelling, Takane & de Leeuw, 1987) but that was not our aim here. Our hypothesis was that LCA would identify distinct groups within the general population that differ in prevalence, and in their perceptions of positive change in social functioning.

## 2 Method

### 2.1 Sample and setting

#### 2.1.1 The MRC 1946 NSHD

The sample comprised participants in the Medical Research Council’s National Survey of Health and Development (NSHD), also known as the British 1946 birth cohort study (Wadsworth, 1991; Wadsworth et al., 1992). Data have been collected at regular intervals since childhood.

The GHQ-28 was completed by survey members at the age 53 follow-up in 1999. At this time the cohort comprised 3673 individuals, excluding those who had already died, those living abroad and permanent refusals. 3035 men and women (83%) were successfully interviewed by research nurses at home visits.

An additional annual survey of women’s health in midlife was undertaken between age 47 and 54 years. Of the original cohort of women 1778 (70%) were included in this additional study, the others had died (6%), refused to take part at earlier follow-ups (12%) or could not be traced (13%). Women participating in the survey were sent annual postal questionnaires.

#### 2.1.2 Analysis sample

Of the 3035 cohort members interviewed in 1999, 2936 (97%) (1440 men and 1496 women) responded to at least one item of the GHQ-28. The sample for this study comprised 2901 individuals (1422 men and 1479 women) who answered all questions. Analyses were conducted separately for men and women due to the observed gender differences in responses to the GHQ (Kilic et al., 1997).

### 2.2 Measures

In the GHQ-28 there are 8 positively worded items, 7 of these comprise the social dysfunction subscale. The remaining positive item (*well and in good health*) appears in the somatic symptoms subscale, with response format equivalent to: [More than usual, the same as usual, less than usual, much less than usual].

We excluded item11 from subsequent analyses (*taking longer over things*), since taking more time is not necessarily a positive social functioning change. This would only be the case if it was interpreted by the survey members as “savouring” the conduct or execution of any experience or activities.

This left us with six out of seven items of the original social dysfunction subscale. We used a positive scoring of the GHQ items that converts Likert scores [1–2–3–4] to a binary outcome [1–0–0–0]. Inspection of the item and response wording confirms that this scoring approach captures positive ratings of change in social functioning (experienced during the last month). We define a positive item as endorsed only if the individual responds with “more than usual” i.e. the first response category, not the others. The exclusion of item 11 from our analyses, should not have any major implications for the generalisability of the social dysfunction subscale, since it was originally designed to capture social dysfunction rather than positive change in social functioning which is of interest here.

### 2.3 Validation of the derived latent classes

The validation of the derived latent classes was performed in two ways. Firstly, internally with respect to a single, a priori chosen, item (GHQ-28, Item 1). This positively worded item enquires general health status (“*well and in good health*”). We also utilised latent factor scores for the remaining GHQ-28 subscales to compare classes in terms of conventional measures of psychological morbidity (somatic symptoms; anxiety/insomnia and severe depression).

Secondly, validation was conducted in relation to external to the GHQ self-report measures. Two measures were used for both men and women, which included job satisfaction (*On the whole how happy would you say you are with your job?*) and a measure of emotional support (*Do you have someone that you can openly share your feelings? Yes/No*). Responses to the question on job satisfaction originally included four categories, (1) Very happy, (2) Fairly Happy (3) Not very happy (4) Unhappy, which were recoded for analyses purposes to a dichotomous variable.

Additional validation items for the women were available from the women’s health survey. Questions on changes in self-confidence and work life over the past 12 months were identified as potential external validation criteria. These questions originally included five response categories, (1) Got a lot better, (2) Got a little better, (3) No change, (4) Got a little worse, (5) Got a lot worse but were recoded to three categories for analyses purposes, (1) Better, (2) Same and (3) negative change.

### 2.4 Statistical modelling: latent class analysis as a model-based cluster analysis

Latent class analysis is the most widely used latent structure model for categorical data (Croudace, Jarvelin, Wadsworth, & Jones, 2003). LCA is a form of cluster analysis which utilises a model-based method i.e. involves the specification of statistical distributions. It differs from more well-known methods such as *K*-means clustering which apply arbitrary distance metrics to group individuals based on their similarity (Everitt, 1992; Everitt & Hand, 1981; Magidson & Vermunt, 2002). LCA derives clusters based on conditional independence assumptions applied to multivariate categorical data distributed as binomial or multinomial variables (Clogg, 1995). Using statistical distributions rather than distance metrics to define clusters helps in evaluating whether a model with a particular number of clusters is able to fit the data, since tests can be performed to observed (*n*_*i*_) versus model expected values (*m*_*i*_) (Read & Cressie, 1988), using exact methods as recommended by Langeheine, Pannekoek, and van de Pol (1996). This comparison gives rise to a *x*^2^ test of global model fit, in which significant values index lack of fit. Here lack of fit means deviation of (model) predicted (*m*) from observed frequencies (*n*). In the parametric bootstrap approach adopted here the model under consideration is evaluated not only in the sample under investigation, but also in a large number (here 10,000) of simulated samples drawn from the probability distributions defined by the estimated model parameters (those returned as the results of the analysis). We report this exact *p*-value and its Monte Carlo standard error (Vermunt and Magidson, 2002), as well as fit criteria based on functions of the maximised log-likelihood (Bozdogan, 1987; Lin & Dayton, 1997). All latent class models were estimated using posterior mode (PM) estimation, implemented through joint Expectation Maximisation and Newton Raphson algorithms, in LatentGOLD 3.0 (Vermunt and Magidson, 2002).

## 3 Results

For men, the bootstrap *p*-values for the likelihood ratio *x*^2^ goodness of fit test returned values of *p* < 0.001 for the one and two classes models (Table 1 Models H_1C_ and H_2C_). For the three class model (H_3C_) the *p*-value increased to *p* = 0.06 (Monte Carlo S.E. 0.002). Information criteria (AIC, BIC, ssaBIC and CAIC) also supported the parsimony of three latent classes identifying population groups with different patterns of reported changes in positive social functioning. For the women participants, the bootstrap *p*-values for the likelihood ratio chi-square goodness of fit test returned values less than 0.001 for the two class model. For the three class model (H_3C_) the *p*-value increased to *p* = 0.29 (Monte Carlo S.E. 0.003) exceeding 0.05 and indicated the fit of the model to the data. As was the case for the men, the information criteria values also supported the parsimony of the three-class solution.

The probability estimates for the three class models for men and women are displayed in Table 2 (these are the latent class indicators, defining the unobserved groups). The class specific conditional probabilities characterise the probabilities of endorsement of each GHQ social item. The unconditional class probabilities (denoted class sizes) indicate the prevalence of the classes – the proportion of the sample classified in each latent class.

### 3.1 Description of the typological (latent) classification

#### 3.1.1 Men

*Class A*_M_ was the modal class (model based prevalence estimate 74.5%). It included men with very low probabilities (*p* < 0.10) of reporting positive changes in social functioning. We refer to this class as “Male-None” signifying absence of or minimal changes in social functioning.

*Class B*_M_ comprised 20.5% of the sample. Men in Class B had a moderate (0.23 < *p* < 0.32) probability of endorsing 4 out of the 6 GHQ items. The highest probabilities were for feeling they were “*playing a useful part*” (more so than usual) and “keeping themselves occupied, more often than usual” (0.31). The lowest probabilities were for feeling they were “doing things well” (0.09) and feeling they were “enjoying daily activities” (0.09). Intermediate probabilities were for feeling “satisfied with tasks, more often than usual” (0.27) and feeling able to make “decisions things” (0.23). We refer to this class as Male-Some signifying some reported positive changes in social functioning.

*Class C*_M_ was the rarest class (5.0%). The men allocated to this class had high (>0.40) probabilities of endorsing the GHQ social items, compared to the other classes. We refer to this class as Male-All signifying positive changes in all areas of social functioning.

#### 3.1.2 Women

Results for women were quite similar to those for men, but differed in the following ways:

*Class A*_*W*_: The first and modal class (model based prevalence estimate 78.6%) comprised women who reported no changes in any area of social functioning. We refer to this group as Women-None and note the slightly higher prevalence compared to men (78.6% versus 74.5%).

*Class B*_*W*_: The second class for women had a model based prevalence estimate of 14.2%. Women in this class felt that they were “keeping occupied more so than usual” (0.56), “playing a useful part, more so than usual” (0.47) and able to make “decisions for things, more often than usual” (0.33). We refer to this class as Women-Some signifying some positive changes in social functioning.

*Class C*_*W*_: The third class for women had a model based prevalence estimate of 7.2%. Women in this class had a high probability of endorsing all of the GHQ positive social items. We refer to this class as Female-All signifying positive changes in all areas of social functioning.

*Allocation to classes*: Assignment of individuals to the categories of the latent class variable was achieved via standard application of Bayes Theorem. Individuals were assigned to the class for which their posterior probabilities of class membership were the highest (modal allocation rule). This enabled us to seek validation of the latent classes as candidate categorical measures of individual differences in a typology of positive social functioning, against other variables.

### 3.2 Validation

#### 3.2.1 Internal validation

Both men (Wald = 60.1, *p* < 0.001) and women (Wald = 74.5, *p* < 0.001) participants allocated to the most positive classes (Male-All C_M_, Female-All C_W_) had the highest probability of reporting that they felt “better than usual” when asked if they were “*well and in good health*” (GHQ Item 1). Those allocated to the most negative classes (Male-None A_M_, Female-None A_W_) had the lowest probability of endorsing that they felt “well and in good health”.

#### 3.2.2 Mean factor scores for somatic symptom, anxiety/insomnia and severe depression scales

**Fig. 1:**
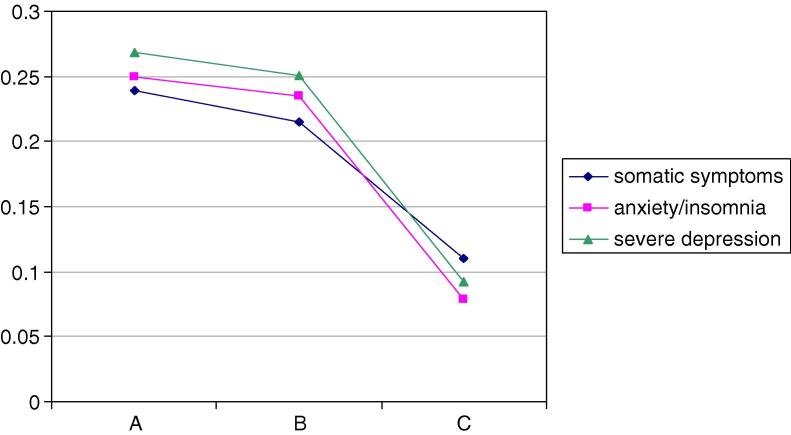
Men participants’ latent class means of GHQ28 factors. (Factor scores (*y*-axis) estimated using Confirmatory Factor Analysis for binary [0–0–1–1] data.)

**Fig. 2:**
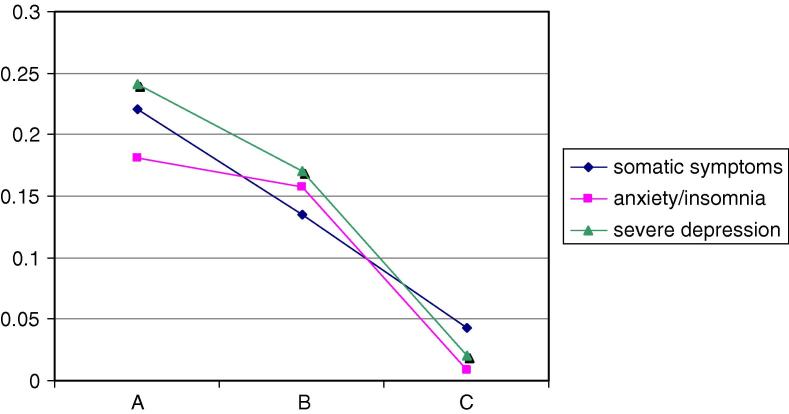
Women participants’ latent class means of GHQ28 factors. (Factor scores (*y*-axis) estimated using Confirmatory Factor Analysis for binary [0–0–1–1] data.)

In men the mean scores for the traditional summary scales from the GHQ-28 varied significantly by latent class: somatic symptoms, Wald = 7.2, *p* < 0.05, anxiety/insomnia Wald = 19.2, *p* < 0.001 and severe depression Wald = 17.6, *p* < 0.001 (see Fig. 1). Men allocated to the most positive latent class (Male-All) had the lowest mean scores on all subscales. In women also the mean scores varied significantly by latent class: somatic symptoms, Wald = 11.1, *p* < 0.05, anxiety/insomnia Wald = 8.8, *p* < 0.05 and severe depression Wald = 12.4, *p* < 0.001 (Fig. 2). Women allocated to the positive class (Women-All, C_M_) had the lowest scores on all subscales.

#### 3.2.3 External validation

##### 3.2.3.1 Men

We observed significant differences between the classes on the item “*On the whole how happy would you say you are with your job*?” (Wald = 6.6, *p* < 0.05) (Table 3). There is a trend that validates our grouping of participants, with the more positive class (C_M_) being the most satisfied with their job, and Class A_M_, the class that reported no positive change in social functioning, getting the least satisfaction from work. We also observed significant differences between the classes on the item “*Overall, do you think you have enough opportunity to talk openly and share your feelings*?” (Wald = 6.5, *p* < 0.05). Class C_M_ (the most positive class) have the highest probability of reporting that they have someone to share feelings with, compared to the other classes.

##### 3.2.3.2 Women

For women we observed significant differences between the classes on changes in self-confidence (Wald = 59.8, *p* < 0.001) and work life, (Wald = 48.4, *p* < 0.001) over the past 12 months. The probability of endorsement of the positive change response option in both items supports our typology, with the most positive class (C_W_) reporting that in the last 12 months they experienced more positive changes on “self-confidence” and “work life” than the other classes.

Furthermore we observed significant differences between the classes on job satisfaction (Wald = 5.1, *p* < 0.05). The trend validates our grouping of women participants, with the more positive class (C_W_) being the most satisfied with their job, and class A_W_ reporting the least satisfaction from work.

We did not observe significant differences between the classes on the opportunity to talk openly and share feelings” (Wald = 0.6, *p* > 0.05). Despite the lack of statistical significance the trend is consistent with our typology. Class C_W_ (the most positive class) have the highest probability of reporting that they have someone to share their feelings, compared to the other classes (see Table 4).

There were no significant associations observed between our latent typology and the demographic characteristics of the participants (results not presented here, available from corresponding author).

## 4 Discussion

We applied latent class analysis to define a social functioning classification, based on 6 of the 7 positive items of the social dysfunction subscale of the GHQ-28. We view social functioning and positive changes in social functioning, as components of general social well-being, as conceptualised by Keyes (1998). Our latent class typology offers a parsimonious summary of patterns of positive responses to GHQ positive items, and captures aspects of social well-being. To our knowledge, it is the first time that positive social functioning change, rather than levels of social functioning per se have been addressed with the GHQ-28.

Our modelling adopts a person centred, rather than a variable centred perspective which focuses on scaling or scoring dimensions. Our analyses indicated that there are three groups, in the UK general population, who differ in the nature of their perceptions of recent changes in positive social functioning. Over three-quarters of men, and women, aged 53 in this nationally representative sample (survivors in a birth cohort study) reported no recent changes in these areas. Of the remainder there were two groups who endorsed either several (three or four), or almost all GHQ social items positively. Our validation analysis revealed differences between these groups for men and women. Gradients in self-reported general health, and other external to the GHQ28 outcomes, such as job satisfaction, sharing feelings and self-confidence, across these three groups were observed. Group membership was also associated with feeling in good health, and scores on the three remaining GHQ-28 factors.

For women, the three classes differed with respect to variables such as self-confidence, perceptions of work life and the opportunity to share feelings. Similarly, the three classes identified from the responses of the male participants, differed with respect to variables such as work satisfaction and the opportunity to share feelings in the expected direction. These additional results seem to rule out the possibility that the derived latent classes (especially the most positive classes) are due to an acquiescent response style, since our typology is validated with variables in a different metric system, derived from items with a dissimilar to the GHQ-28 response format.

Our results have implications for the future use of the GHQ-28 by applied researchers. Apart from the four summary scores that can be obtained for each of the GHQ-28 subscales, we offer a further novel analysis that creates groupings from a sub-set of questions from this popular instrument. Applying latent class analysis to the social dysfunction items, recoded in the manner suggested by this paper, will provide GHQ users with another outcome – positive change in social functioning – that has relevance in the general population and clinical samples. This new approach to analysis of the GHQ data may be of particular interest to researchers who evaluate the effectiveness of interventions that target social functioning as well as other outcomes. We recognise that in this report, the models have been estimated using commercial software, but they can also be estimated with software freely available on the internet such as LEM (Vermunt, 1997) or LATCLASS (Bartholomew & Knot, 1998).

Strengths of the study include the population based nature of the sample, comprising a general population birth cohort. The NSHD participants are homogenous for age, and so age cannot have influenced our findings, however they may not generalise to populations of different ages. Our analysis capitalised on an under-used feature of GHQ responses, and therefore our treatment of this instrument is quite distinct from other recommended scoring methods. We justify this through our interest in positive health outcomes rather than morbidity per se.

## Figures and Tables

**Table 1:** Latent class analysis results

		LL	L^2^	BIC(L^2^)	AIC(L^2^)	ssaBIC	CAIC	df	*p* value^a^ for LR chi-square	Monte Carlo S.E.	Classification error
*Men n* *=* *1422*											
Model 1: Single class	H_1C_	−2720.3	859.4	445.3	745.4	5445.7	389.5	57	.0000	.0000	.000
											
Model 2: Two classes	H_2C_	−2343.2	105.1	−258.1	5.1	4719.6	−307.4	50	.0001	.0001	.023
											
Model 3: Three classes	H_3C_	−2321.5	61.7	−250.7	−24.3	4704.4	−293.4	43	.0619	.0024	.113
											
*Women n* *=* *1479*											
Model 1: Single class	H_1C_	−2911.1	935.5	519.0	821.5	5793.3	456.1	57	.0000	.0000	.000
											
Model 2: Two classes	H_2C_	−2514.4	142.1	−223.2	42.1	5037.4	−270.6	50	.0000	.0000	.036
											
Model 3: Three classes	H_3C_	−2469.1	51.4	−262.8	−34.6	4975.1	−303.6	43	.2888	.0028	.069

*Abbreviations*: AIC: Akaike Information Criterion; BIC: Bayesian Information Criterion; ssaBIC: sample size adjusted BIC; CAIC: consistent AIC.

a Bootstrap *p*-values from 10,000 simulations.

**Table 2:** Prevalence estimates and class-specific response probabilities

	Men			Women		
	Class A_M_	Class B_M_	Class C_M_	Class A_W_	Class B_W_	Class C_W_
Prevalence: Class size %	74.5%	20.5%	5.0%	78.6%	14.2%	7.2%
Label	“None (A_M_)”	“Some (B_M_)”	“All (C_M_)”	“None (A_W_)”	“Some (B_W_)”	“All (C_W_)”
						
GHQ10 “busy and occupied”	0.11	*0.31*	**0.46**	0.10	**0.56**	**0.44**
						
GHQ12 “doing things well”	0.01	0.09	**0.75**	0.01	0.05	**0.59**
						
GHQ13 “satisfied with tasks”	0.02	*0.27*	**0.94**	0.02	0.01	**0.99**
						
GHQ14 “useful part in things”	0.01	*0.32*	**0.84**	0.01	**0.47**	**0.76**
						
GHQ15 “decisions things”	0.01	*0.23*	**0.72**	0.02	*0.33*	**0.47**
						
GHQ17 “enjoy activities”	0.02	0.09	**0.43**	0.03	0.19	*0.31*

*Italic* = 0.2 < *p* < 0.4. Bold = 0.4 < *p* < 1.00.

**Table 3:** Latent classes validation and corresponding Wald tests: men

	*N*	A_M_	B_M_	C_M_	Wald	*p*-value
*GHQ-28: Item* 1 – *Well and in good health?*						
Same/less/much less than usual	1305	97%	84%	61%	60.1	*p* < 0.001
More than usual	117	3%	16%	39%		
						
*How happy would you say you are with your job?*						
Happy	202	83%	88%	97%	6.6	*p* < 0.05
Unhappy	1207	17%	12%	3%		
						
*Enough opportunity to talk openly and share feelings?*						
No	1265	9%	17%	5%	6.5	*p* < 0.05
Yes	140	91%	83%	95%		

**Table 4:** Latent classes validation and corresponding Wald tests: women

	*N*	A_W_	B_W_	C_W_	Wald	*p*-value
*GHQ-28: Item* 1 – *Well and in good health?*						
Same/much less than usual/	1311	94%	75%	64%	74.5	*p* < 0.001
More than usual	168	6%	25%	36%		
						
*Self-confidence*^a^						
Positive change	244	15%	38%	59%	59.8	*p* < 0.001
No change	688	67%	54%	34%		
Negative change	172	18%	8%	7%		
						
*Worklife*^a^						
Positive change	212	14%	34%	46%	48.4	*p* < 0.001
No change	636	62%	42%	38%		
Negative change	256	24%	24%	16%		
						
*How happy would you say you are with your job?*						
Happy	1296	88%	93%	96%	5.1	*p* < 0.05
Unhappy	146	12%	7%	4%		
						
*Enough opportunity to talk openly and share feelings?*						
No	1273	14%	16%	12%	0.6	NS
Yes	189	86%	84%	88%		

NS – non significant.

a Items from the Women Health Survey.
